# An Efficient Hairy Root System for Validation of Plant Transformation Vector and CRISPR/Cas Construct Activities in Cucumber (*Cucumis sativus* L.)

**DOI:** 10.3389/fpls.2021.770062

**Published:** 2022-02-11

**Authors:** Doai Van Nguyen, Trang Thi-Huyen Hoang, Ngoc Thu Le, Huyen Thi Tran, Cuong Xuan Nguyen, Yong-Hwan Moon, Ha Hoang Chu, Phat Tien Do

**Affiliations:** ^1^Laboratory of Plant Cell Biotechnology, Institute of Biotechnology, Vietnam Academy of Science and Technology, Hanoi, Vietnam; ^2^Department of Integrated Biological Science, Pusan National University, Busan, South Korea; ^3^Division of Plant Sciences, University of Missouri, Columbia, MO, United States; ^4^Department of Molecular Biology, Pusan National University, Busan, South Korea; ^5^Graduate University of Science and Technology, Vietnam Academy of Science and Technology, Hanoi, Vietnam

**Keywords:** cucumber, hairy root, *Rhizobium rhizogenes*, CRISPR/Cas9, root hair-less, *CsbHLH66*, *CsbHLH82*, *ROOTHAIRLESS1*

## Abstract

Hairy root induction system has been applied in various plant species as an effective method to study gene expression and function due to its fast-growing and high genetic stability. Recently, these systems have shown to be an effective tool to evaluate activities of CRISPR/Cas9 systems for genome editing. In this study, *Rhizobium rhizogenes* mediated hairy root induction was optimized to provide an effective tool for validation of plant transformation vector, CRISPR/Cas9 construct activities as well as selection of targeted gRNAs for gene editing in cucumber (*Cucumis sativus* L.). Under the optimized conditions including OD_650_ at 0.4 for infection and 5 days of co-cultivation, the highest hairy root induction frequency reached 100% for the cucumber variety Choka F1. This procedure was successfully utilized to overexpress a reporter gene (*gus*) and induce mutations in two *Lotus japonicus ROOTHAIRLESS1* homolog genes *CsbHLH66* and *CsbHLH82* using CRISPR/Cas9 system. For induced mutation, about 78% of transgenic hairy roots exhibited mutant phenotypes including sparse root hair and root hair-less. The targeted mutations were obtained in individual *CsbHLH66*, *CsbHLH82*, or both *CsbHLH66* and *CsbHLH82* genes by heteroduplex analysis and sequencing. The hairy root transformation system established in this study is sufficient and potential for further research in genome editing of cucumber as well as other *cucumis* plants.

## Introduction

Originating in the Indian subcontinent, cucumber (*Cucumis sativus* L.) has become one of the top consumed vegetables widely grown in the world (Paris et al., 2012; Taha et al., 2020). However, cucumber cultivation currently has suffered from biotic and abiotic stresses (Liu et al., 2013; Chojak-Koźniewska et al., 2018; Fan et al., 2020; Taha et al., 2020). There are various approaches used to improve cucumber production such as optimizing cultivation conditions (Taha et al., 2020), plant grafting (Elsheery et al., 2020), and breeding. Of which, modem breeding with the help of genetic engineering has been known as the most effective method because genetic variability of cucumber varieties is low 3–8% (Chee, 2001; Plader et al., 2007). The genome editing boosted by CRISPR/Cas systems has been widely applied on various crops as a powerful tool to improve plant traits and gene function studies (Zhang et al., 2021). In cucumber, there have been several reports of using CRISPR/Cas9 for targeted gene knockout (Chandrasekaran et al., 2016; Hu et al., 2017). However, applications of genome editing in cucumber have been challenged by less effective transformation procedures that are time consuming and labor extensive processes (Hu et al., 2017). Therefore, the improvement of procedures and the development of supporting tools are necessary to accelerate genetic engineering in cucumber.

*Rhizobium rhizogenes*-mediated hairy root cultures have been established and developed in different plant species for biomass, recombinant protein production, gene function studies, and secondary metabolite researches (Gutierrez-Valdes et al., 2020). In addition, hairy roots also could be used as materials for generating whole plants in certain reports (Trulson et al., 1986; Bueno dos Reis et al., 2007; Butler et al., 2020; Zhang et al., 2020). Recently, the hairy root transformation has been widely utilized to validate and optimize induced mutagenesis by the CRISPR/Cas9 system (Michno et al., 2015; Du et al., 2016; Jacobs and Martin, 2016; Li et al., 2019; Le et al., 2020). Both *in vitro* and *in vivo* cucumber hairy root cultures have been established for different research purposes (Shi and Lindemann, 2006; Anuar et al., 2011; Fan et al., 2020). However, the application of cucumber hairy root transformation for genome editing using CRISPR/Cas systems has not been reported yet. Moreover, it has been reported that hairy root induction frequency is affected by cucumber varieties, bacterial strains, and transformation methods (JingLong et al., 2017; Fan et al., 2020).

In this study, an efficient procedure for *in vitro* cucumber hairy root induction and transformation was established for a local cucumber cultivar. This system was also applied for the expression of a reporter gene (*gus*) as well as validation of CRISPR/Cas construct activity. The targeted mutations including heterozygous, homozygous, and biallelic indels in two targeted genes *CsbHLH66* and *CsbHLH82* was obtained at primary and propagated transgenic hairy roots. These results provide an effective tool for gene expression, gene function, and genome editing studies in cucumber.

## Materials and Methods

### Plant Material

Seeds of a local cucumber (*Cucumis sativus* L.) cultivar Choka F1 were surface sterilized in 15% commercial bleach (bleaching solution containing 5% sodium hypochlorite, Duc Giang, VN) for 15 min, then washed 4–5 times in sterile water (Hoang et al., 2021). The sterilized seeds were soaked in autoclaved water for 3 h at 26°C. After removing seed coats, unwounded seeds were placed on MS30 medium (Table 1) for 2 days in the dark.

**Table 1:** The list of media used in cucumber hairy root transformation.

Medium	Composition or references
MS	Murashige and Skoog, 1962
MS30	MS, 30 g/L sucrose and 7.5 g/L agar; pH 5.8
Re-suspension	0.5X MS, 30 g/L sucrose and 200 μM/L AS; pH 5.8
Co-cultivation	MS30, 200 μM AS, pH 5.8
Hairy root culture	MS30, 300 mg/L cefotaxime; pH 5.8
Hairy root selection	Hairy root culture medium added selective agents

AS, antibiotics and selective agents were added to medium after autoclaving.

### CRISPR/Cas9 Vector Construction for Hairy Root Assay

The *CsbHLH66* and *CsbHLH82* and other related protein sequences were discovered by the BlastP (NCBI) using the *Lotus japonicus* ROOTHAIRLESS1 (LjRHL1) protein sequence (Karas et al., 2009) as a reference. To induce targeted mutations in *CsbHLH66* and *CsbHLH82* genes, gRNAs were generated by the CCTop program (Stemmer et al., 2015). The CRISPR/Cas9 plant transformation vectors were constructed based on the pKSE401 vector (addgene plasmid # 62202), a gift from Qi-Jun Chen laboratory (Xing et al., 2014), in which, the *neomycin phosphotransferase II* (*ntpII*) gene was replaced by the *basta resistance* (*bar*) gene. The coding sequence of *bar* gene was amplified from the binary vector pZY102 (Zeng et al., 2004) using primer pairs: *Nco*I-*bar*-F and *Rsr*II-*bar*-R (Supplementary Table S1). The PCR amplifications were conducted using the Phusion High-Fidelity DNA Polymerase kit (Cat. No. F553L – Thermo Scientific) following the manufacturer’s instructions. After purification, the PCR products were inserted into pKSE401 at *Nco*I and *Rsr*II sites. For gRNA insertion, *Bsa*I-*CsbHLH66* gRNA-gRNA scaffold-*U6* terminator-*Bsa*I and *Bsa*I-*U6* promoter-*CsbHLH82*-gRNA-*Bsa*I fragments were generated from the pKSE401 template by PCR using the listed primers (Supplementary Table S1). These two fragments were assembled into the destination vector pKSE401 using *Bsa*I enzyme sites. The final constructed vector was confirmed by sequencing and named pKSE401-CsbHLH82-66. The pKSE401-CsbHLH82-66 and pZY102 binary vectors were mobilized into *R. rhizogenes* strain K599 and used for cucumber hairy root transformation.

### *Rhizobium rhizogenes*-Mediated Transformation and Hairy Root Induction

*Rhizobium rhizogenes* strains including ATCC 15834 (pRi15834) and K599 (pRi2659) wild-type or carrying binary vectors were used for bacterial mediated transformation. Briefly, single colonies were cultured in 5 ml liquid yeast extract peptone (YEP) medium supplemented with appropriate antibiotics (25 mg/L rifampicin for the *R. rhizogenes* strain K599; no antibiotic for the *R. rhizogenes* strain ATCC 15834; 25 mg/L rifampicin plus 50 mg/L kanamycin the *R. rhizogenes* strain K599 harboring pKSE401-CsbHLH82-66 vector; 25 mg/L rifampicin plus 100 mg/L spectinomycin for the *R. rhizogenes* strain K599 haboring pZY102 vector). The culture was shaken at 28°C and 200 rpm for 4–6 h, transferred to 100 ml of fresh medium and cultured overnight until OD_650_ reached 0.8–1.0. On the next day, the cells were harvested by centrifugation at 3,000 rpm for 15 min and re-suspended in the resuspension medium (Table 1) to get the OD_650_ of 0.4.

**Figure 1:**
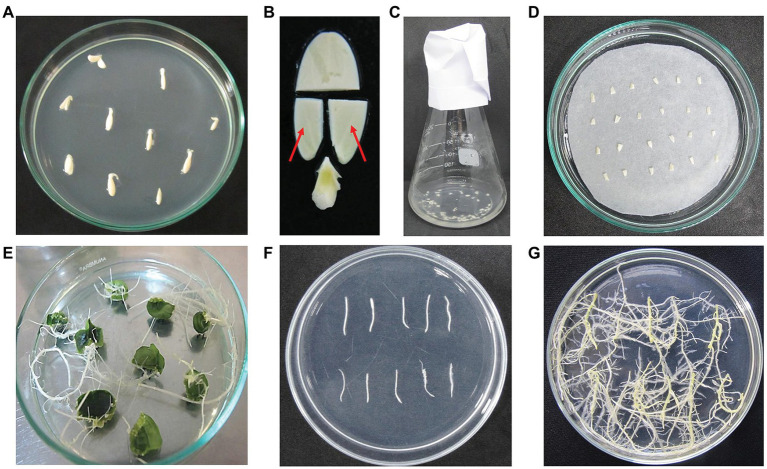
Main steps of cucumber hairy root induction. **(A)** The 2-day-old seedlings; **(B)** cotyledon explants preparation. The red arrows indicate the cotyledon fragments used for bacterial infection; **(C)** cotyledon fragments were soaked in bacterial suspension; **(D)** the infected explants on co-cultivation medium; **(E)** hairy root formation at 20 days after co-cultivation; **(F)** hairy root fragments were transferred to a new medium; **(G)** hairy roots developed on hairy root culture medium.

The cotyledons from 2-day-old seedlings were collected and used as explants for infection. The distal parts and proximal petioles were excised and removed. Then, the proximal parts of the cotyledons were cut in half along the midrib to get small pieces (Figures 1A,B). Cotyledon segments were kept on wet filter papers during the cutting time to avoid water loss. About 60–70 explants were soaked in 30 ml bacterial suspension for 30 min at room temperature (Figure 1C). Then, explants were removed from the extra liquid and placed on the co-cultivation medium (Table 1) covered by a filter paper (Whatman # 1001-090) on top. The culture plates were kept at 25 ± 2°C in the dark for 5 days. Next, explants were washed by autoclaved water, blotted by sterile filter papers, and placed on the hairy root culture medium (Table 1) for 15–20 days. Root tip fragments (2 cm) were cut and transferred to fresh medium for elongation and development. Transgenic hairy roots were obtained on the hairy root selection medium (Table 1) for 10–15 days (Figure 1E). Cucumber hairy root induction and selection were conducted at 25 ± 2°C under a light density of 2,000 lux with a photoperiod of 16/8 h.

### GUS Histochemical Analysis

The pZY102 transformed hairy roots survived on the selection medium were collected for GUS histochemical analysis using 5-bromo-4-chloro-3-indolyl glucuronide buffer at 37°C for 16 h (Jefferson et al., 1987). GUS expression in cucumber hairy roots was observed and captured by a regular camera.

### Identification and Characterization of Induced Mutant Hairy Roots

Root hair phenotypes were analyzed using the method described by Karas et al. (2005, 2009), in which root hairs were observed under ZEISS Stemi 305 stereo microscope and captured with AxioCam ERc 5 s digital camera. Genomic DNA was extracted from hairy roots using the CTAB method (Doyle and Doyle, 1987). Hairy roots with induced mutations in *CsbHLH* genes were detected by heteroduplex analysis on polyacrylamide gel electrophoresis (PAGE; Chen et al., 2012) with some modifications. Briefly, the spanning target regions on the *CsbHLH* genes were amplified using flanking specific primers (Supplementary Table S1). The PCR amplifications were conducted using DreamTaq Green PCR Master Mix (Cat. No. K1081 – Thermo Scientific) with the following conditions: initial denaturation at 94°C for 4 min; 30 cycles at 94°C for 20 s, 55°C for 20 s and 72°C for 30 s, and a final extension at 72°C for 5 min. The mixture containing equal amplicons from wild type (WT) and transgenic hairy roots was denatured at 95°C for 10 min and slowly cooled down to room temperature to form heteroduplexes. The heteroduplexes were detected by PAGE using 12% polyacrylamide gel. The PCR amplicons were further purified and ligated to the pJET1.2 cloning vector (Thermo FisherScientific, United States) for sequencing. The sequencing was performed on the ABI3500XL system (Applied Biosystems). *CsbHLH* mutant sequences were compared to wild-type sequences using BioEdit 7.2.

### Statistical Analysis

All statistical analyses were performed using SPSS software version 24.1. The significant analysis (value of *p* < 0.05) between experiments was performed by Student’s *t*-test or one-way ANOVA followed by post-hoc Duncan’s new multiple range tests.

## Results

### Optimization of Cucumber Hairy Root Induction Procedure

**Figure 2:**
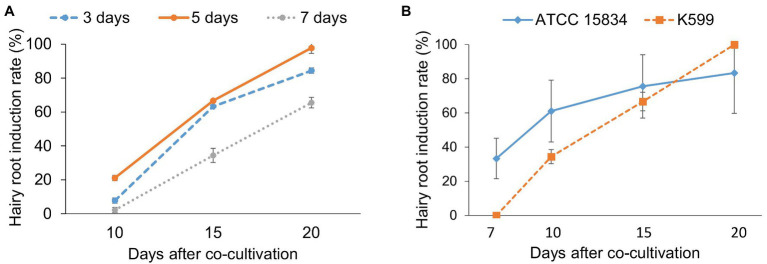
The effects of co-cultivation duration and bacterial strains on cucumber hairy root induction. **(A)** Co-cultivation time and hairy root induction. **(B)** *Rhizobium rhizogenes* strains affect hairy root induction. Hairy root induction was observed and counted at different time points. Data are presented as mean ± SD of three replicates. About 30 explants were used for each treatment.

In this study, cotyledon pieces from 2-day-old seedlings of ChokaF1 cultivar were first inoculated with *R. rhizogenes* K599 strain to evaluate the effects of co-cultivation duration on induced hairy root efficacy. After 3, 5, and 7 days of co-culture, explants were transferred onto hairy root induction medium. There were 21.1 ± 1.6% explants of the 5-day co-cultivation treatment that showed hairy root formation at 5 days on induction medium, and it dramatically reached 100% at 20 days after co-cultivation (Figure 2A). While the highest rate of explants in 3- and 7-day co-cultivation producing hairy roots after 20 days were 84.4 ± 1.6% and 65.6 ± 3.14%, respectively, and no more explants produced hairy roots if having the culture period prolonged. Bacterial overgrowth was also observed in the 7-day co-cultivation treatment (data not shown). Therefore, 5-day co-cultivation was sufficient for inducing hairy roots from the local cucumber ChokaF1 and further used for all cucumber hairy transformation experiments.

The effects of *R. rhizogenes* strain (ATCC 15834), a widely used *R. rhizogenes* strain to induce hairy roots in various plant species including cucumber (Shi et al., 2006; Shi and Lindemann, 2006), were also evaluated on the local cucumber cultivar in comparison to the K599 strain (Figure 2B). In this experiment, explants inoculated with ATCC 15834 strain produced hairy roots earlier than those infected with the K599 strain. About 33.3 ± 11.9% explants induced hairy roots (2–3 cm) after 1 week on hairy root induction medium, whereas no hairy root was observed from explants infected with K599. After 10 days, the rate of explants producing hairy roots was 34.4 ± 4.2% for K599, while it was double (61.1 ± 18.1%) for the ATCC 15834 strain. The number of ATCC 15834 inoculated explants generating hairy root was continuously higher than K599 treated ones after 15 days. However, after 20 days the highest hairy root induction frequency from ATCC 15834 infected explants was lower (83.3 ± 16.6%) than the ones from K599 (100%). In addition, the formation of calli were observed from wounding sites as well as whole explants which were inoculated with the ATCC 15834 strain. Whereas very few calli formed from K599 inoculation (Supplementary Figure S1).

**Figure 3:**
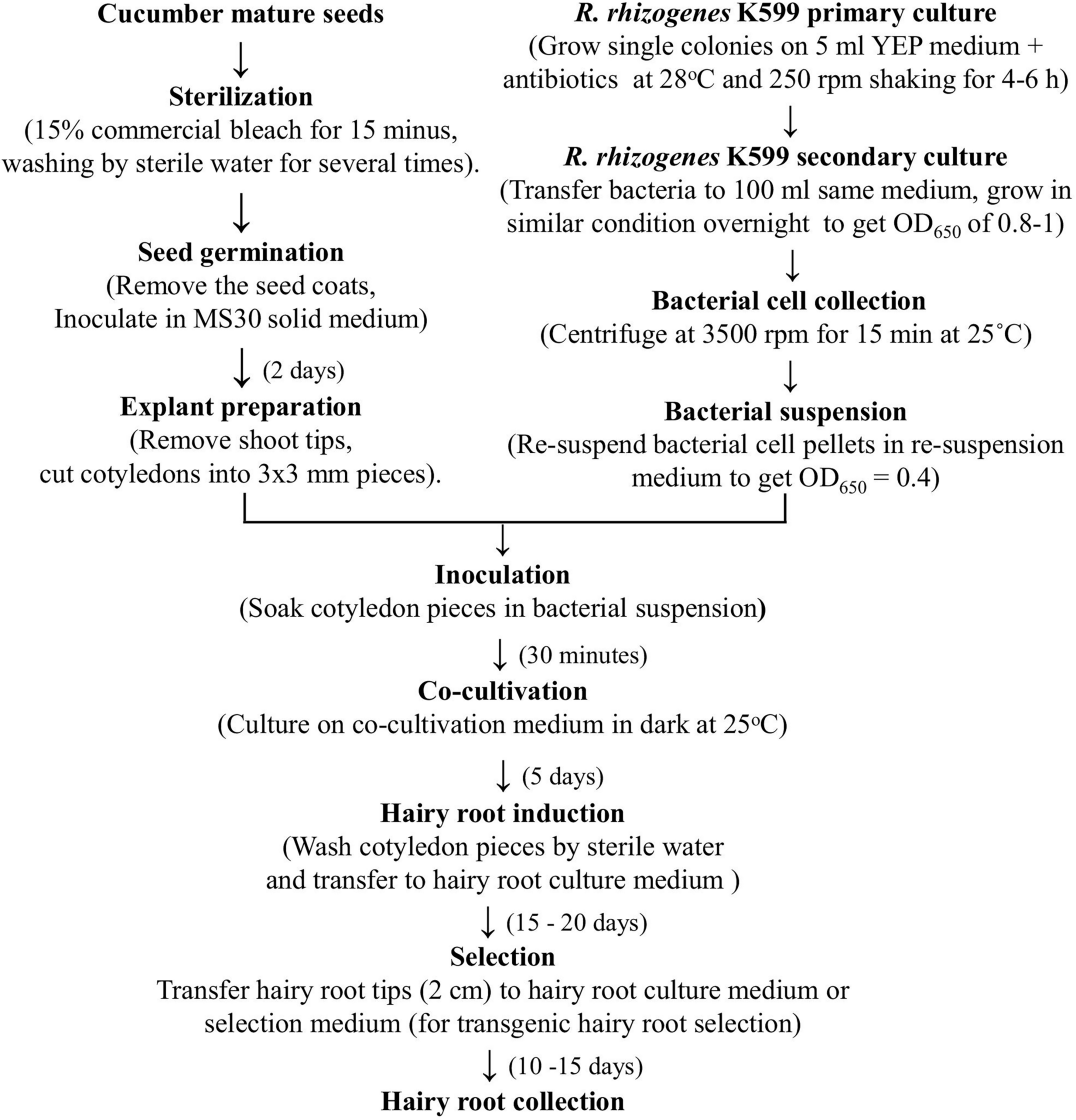
The procedure for cucumber hairy root induction using cotyledons.

From all the above results, an optimized procedure for cucumber *in vitro* hairy root induction was established, in which cotyledons of 2-day seedlings were infected with *R. rhizogenes* K599 and co-cultivated for 5 days. The total time to conduct the procedure is less than 1 month and the hairy root induction rate could reach up to 100%. Details of the procedure are presented in Figures 1, 3.

### Utilizing Cucumber Hairy Root System for Validating Plant Transformation Vector Activities and Transgene Expression

**Figure 4:**
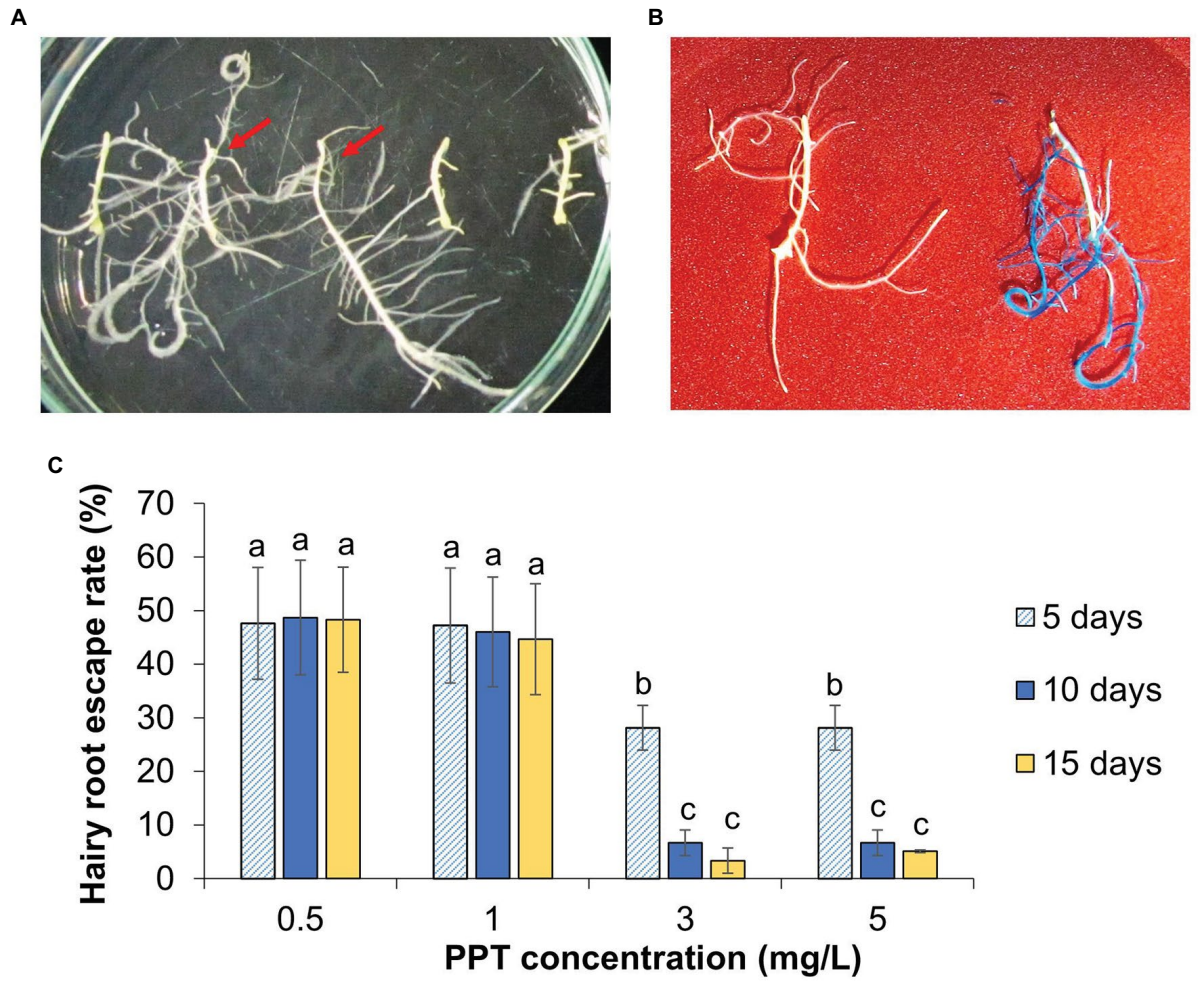
The transgenic hairy root selection and histochemical analysis. **(A)** Hairy roots at 10 days on selection medium supplemented with 3 mg/L PPT. The red arrows indicate the PPT resistant hairy roots. **(B)** GUS histochemical analysis of non-transgenic (left) and transgenic hairy roots (right). **(C)** Effect of PPT concentrations on the selection efficiency. The escaped hairy roots are referred to the PPT resistant hairy roots without GUS staining. Data are presented as mean ± SD of three replicates. Different letters indicate significant differences based on one-way ANOVA analysis with Duncan’s test (*p* < 0.05, *n* = 30).

Utilizing the developed hairy root system, the expression of transgenes was conveniently assessed. K599 strain harboring binary vector pZY102 carrying a reporter gene (*gus*) and selective gene (*bar*) was transferred into cucumber cotyledon pieces. The induced hairy roots (2 cm) were removed from explants and cultured on the selection medium containing herbicide phosphinothricin (PPT) concentrations ranging from 0.5 to 5 mg/L. The herbicide-resistant hairy roots showed normal morphology and development, while non-resistant hairy roots were completely inhibited and turned yellow without elongation (Figure 4A). The analysis of *gus* gene expression in putative transgenic hairy roots (herbicide resistant ones) after 10 days on selection medium indicated that not all of them were positive for GUS staining (Figures 4B,C; Supplementary Figure S2). The number of selection-escape hairy roots was varied and depended on PPT concentration. On the medium with 3 or 5 mg/L PPT, 6.7 ± 1.6% of survived hairy roots escaped. This number raised up to 46 ± 7.2% and 48.7 ± 7.6% in the medium containing 1.0 and 0.5 mg/L PPT, respectively. On the 15th day in selection medium of 3 mg/L PPT, the number of GUS positive/negative hairy roots was not significantly different from hairy roots on the 10th day (Figure 4C). This result indicates that transgenic hairy roots could be effectively obtained in 3 mg/L PPT medium in 10 days with the lowest rate of escaping from the selection. Therefore, the activity of the binary vector and the expression of transgenes could be effectively analyzed using the cucumber *in vitro* hairy root system for 10–15 days.

### Identification of Targeted Genes for CRISPR/Cas9 Induced Mutagenesis of Cucumber Hairy Root

**Figure 5:**
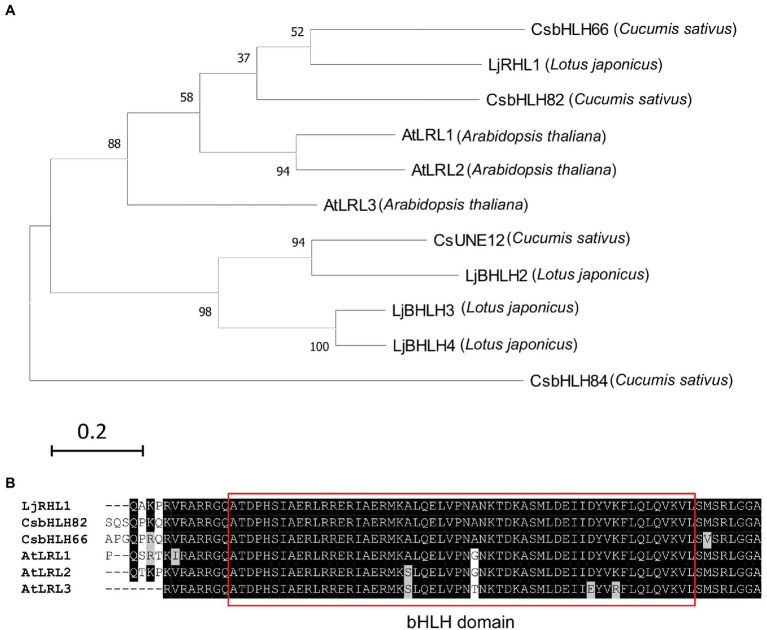
The phylogenetic tree and sequence analysis of the LjRHL1 homologs. **(A)** Molecular phylogenetic tree of LjRHL1 protein sequence and its homologs were conducted by MEGA X using the maximum likelihood method and JTT matrix-based model. Numbers at nodes indicate the percentage bootstrap scores from 1,000 replicates. The scale bar represents 0.2 estimated number of amino acid substitution events per site. **(B)** Sequence alignment bHLH domain (in red box) of LjRHL1 in *Lotus japonicus* and its homologs in cucumber and Arabidopsis.

In addition to testing the transgene expression, the developed hairy root induction system in this study was applied for genome editing studies utilizing the CRISPR/Cas9 system. Four candidate sequences, namely, CsbHLH82, CsBHLH66, CsUNE12, CsBHLH84, were identified when the LjRHL1 protein sequence (from *Lotus japonicus*, Karas et al., 2009) was used as a reference for searching on cucumber database (NCBI: ASM407v2) by protein BLAST tool. The phylogenetic tree was constructed using the four candidate sequences, the LjRHL1 sequence, and 3 ortholog sequences from *Arabidopsis thaliana* by the maximum likelihood method and JTT matrix-based model in MEGA X (Kumar et al., 2018). The result indicated that CsbHLH82 and CsbHLH66 were highly close to LjRHL1 (Figure 5A). Moreover, the bHLH domain sequences of the CsbHLH82 and CsbHLH66 were identical to the one of the LjRHL1 (Figure 5B). Therefore, both *CsbHLH82* and *CsbHLH66* were selected as the targets for mutagenesis in the cucumber hairy roots.

### Inducing Targeted Mutations in Cucumber Hairy Roots by CRISRP/Cas9 System

**Figure 6:**
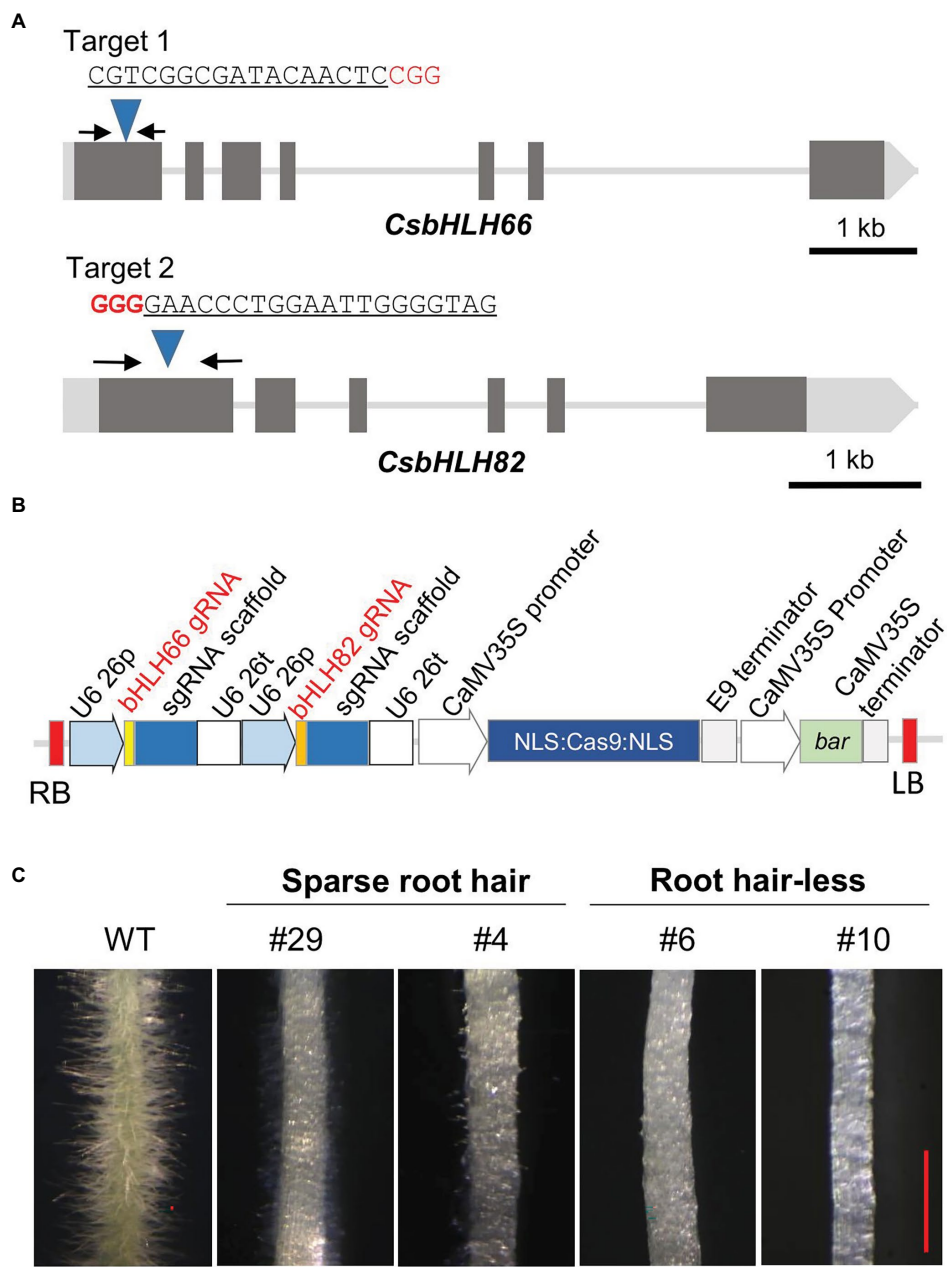
CRISPR/Cas9 construction and phenotyping of mutant hairy roots. **(A)** Maps of *CsbHLH66*, *CsbHLH82* genes and target locations. The black arrows indicate primer binding sites for genotyping. **(B)** T-DNA regions of the CRISPR/Cas9 plant transformation vector. **(C)** Root hair phenotypes of WT and mutant hairy roots. The red scale bar indicates 1 mm.

The DNA sequences of both *CsbHLH82* and *CsbHLH66* genes were reconfirmed in the local cucumber variety for developing a CRISPR/Cas9 vector. Two gRNAs targeting the first exon of *CsbHLH66* and *CsbHLH82* were identified using the CCTop tool (Figure 6A) and inserted in the CRISPR/Cas9 expression binary vector (Figure 6B) with the purpose to induce individual and simultaneous targeted mutations of the two tested genes.

Targeted mutations of *CsbHLH82* and *CsbHLH66* were induced in hairy roots using the K599 strain that harbored the CRISPR/Cas9 vector. Each induced hairy root considered as an independent line was transferred to the selection medium. Phenotyping based on more than 80 hairy root lines indicated that there were three morphological groups (Figure 6C; Supplementary Figure S3) including (1) abundant root hairs like wild-type hairy roots (22.04%), (2) sparse and short root hairs (52.54%), and (3) root hair-less (25.42%).

**Figure 7:**
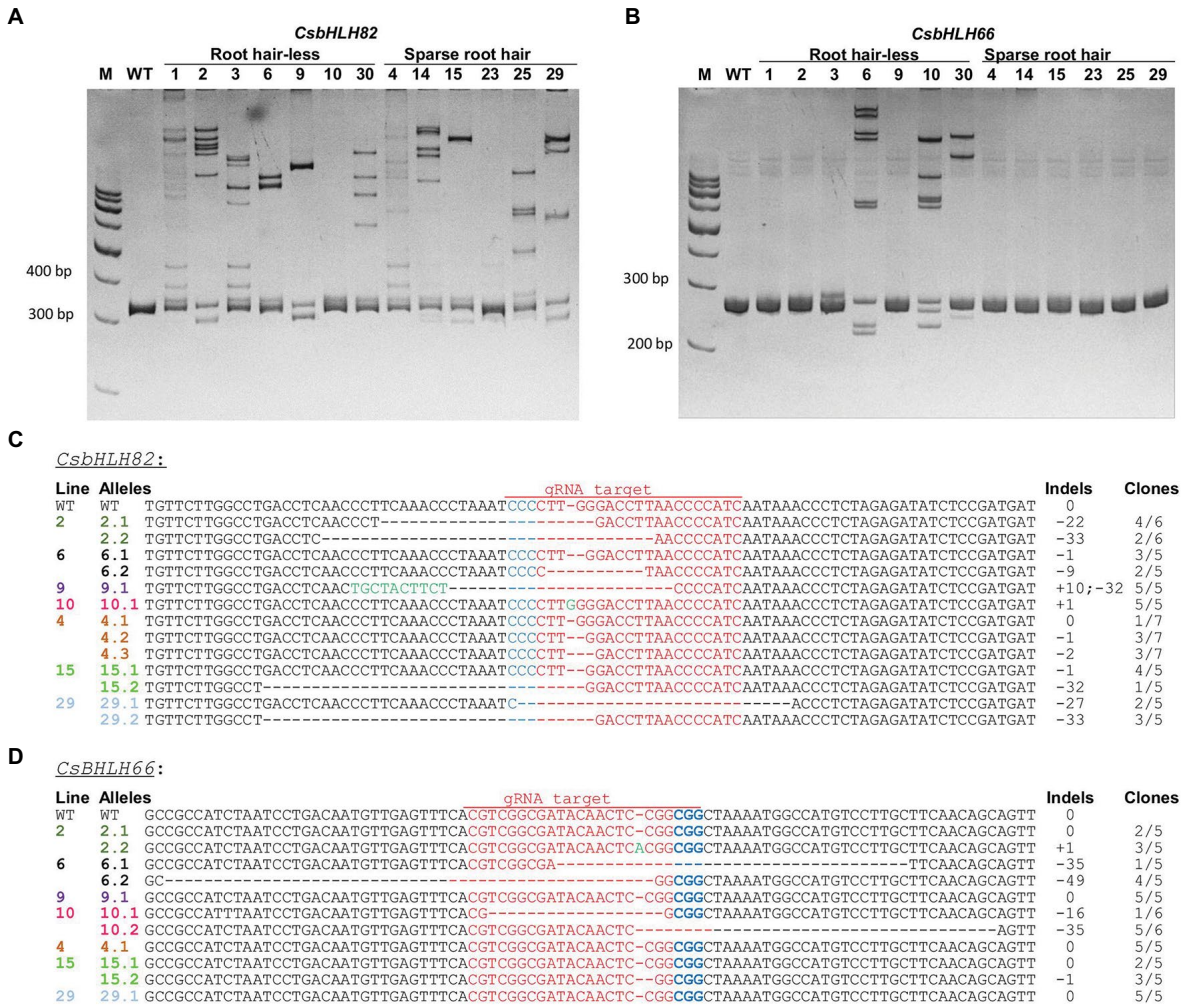
Identification and characterization of induced mutations in cucumber hairy roots. **(A,B)** Heteroduplex analysis of *CsbHLH82* and *CsbHLH66*, respectively. M: 100 bp DNA Ladder; WT: wild type. **(C,D)** Expanding targeted sequences of *CsbHLH82* and *CsbHLH66* genes. Red letters indicate target sequences. Blue letters refer to PAM sequences. Green letters indicate inserted nucleotides. The indels indicate targeted sequence changes: 0 for no change, − for deletion, + for insertion. The number of clones used for sequencing was indicated.

Heteroduplex analysis of 13 randomly selected hairy root lines from group 2 and 3 (Figures 7A,B) showed that all tested lines contained mutations on *CsbHLH82* upon the shifted DNA band observation on the PAGE gel as compared to the wild type hairy roots (Figure 7A). In the case of *CsbHLH66*, only 4 hairy root lines (3, 6, 10, 30) exhibited induced mutations (Figure 7B). Importantly, these 4 lines all belonged to group 3 having the root hair-less phenotype.

The induced mutations in *CsbHLH* genes of 3 lines (4, 15, 29) from the spare root hair group and 4 lines (2, 6, 9, 10) from the root hair-less group were further characterized *via* sequencing targeted amplicons (Figures 7C,D). For the *CsbHLH82* gene, induced mutations occurred in all tested hairy root lines (Figure 7C). Various types of indels were observed at the target locations, in which the deletion sizes ranged from −1 bp up to −33 bp and the insertions were from +1 to +10 bp. Different from *CsbHLH82*, targeted mutations in *CsbHLH66* were confirmed in only 5 of 7 tested root hair lines, including 2 mutant alleles in lines 2 and 15, which were undetectable in heteroduplex analysis because of only 1 bp indels (1 bp insertion or deletion). The 1 bp insertion and big deletions (−16 to −49 bp) in the *CsbHLH66* targeted gene were found in 3 tested hair-less root lines, while 1 bp deletion was found in one sparse root hair line 15 only (Figure 7D). Moreover, two mutant alleles (biallelic mutation) of targeted genes were also observed in many hairy root lines such as lines 6 and 10 for *CsbHLH66* gene and lines 2, 6, 15, and 29 of *CsbHLH82* gene (Figures 7C,D). The homozygous mutant allele was found in line 9 (for *CsbHLH82* gene). Three different alleles (chimeric alleles) of the *CsbHLH82* gene were also observed in hairy root line 4. Importantly, lines 6 and 10 contained null mutations of both the *CsbHLH82* and *CsbHLH66* genes. As a consequence, it is obvious that our developed hairy root system has a great potential for genome editing studies in cucumber.

The stability of induced mutations of targeted genes was further confirmed in *in vitro* propagated hairy roots. The root fragments from line 6 and 10 were cut and propagated in a new medium for the next generations. Heteroduplex, Sanger, and amplicon sequencing analysis were conducted using DNA extracted from the third propagation root lines. DNA band patterns in PAGE and sequencing data indicated that the mutations observed in the propagated lines were the same as in the primary roots (first propagation) for both *CsbHLH82* and *CsbHLH66* (Supplementary Figure S4). This result demonstrated that stably induced mutations were conducted in cucumber hairy roots.

## Discussion

### Establishment of an Efficient Procedure for Cucumber *in vitro* Hairy Root Induction

In this study, an efficient procedure for cucumber *in vitro* hairy root induction was established. The hairy root induction frequency could reach 100% after 20 days of co-cultivation using the *R. rhizogenes* K599 strain. Different factors including explants, co-inoculation, and co-cultivation conditions have been reported to influence plant transformation efficacy as well as hairy root induction. In our results, the 5 days co-cultivation was selected because of the highest hairy root induction efficiency. Co-cultivation duration extended to 7 days negatively influenced cucumber hairy root formation due to the overgrowth of bacteria after inoculation. This result is in agreement with Shi et al. (2006), in which 5 days of co-cultivation was considered to be optimal for cucumber *in vitro* hairy root induction. One other optimization in this study was the use of cotyledon pieces from 2-day-old seedlings as initial explants instead of 10-day-old seedlings as in previous studies (Shi et al., 2006; Shi and Lindemann, 2006) for hairy root generation. Such kind of explants was reported to be optimal for cucumber transformation and regeneration only (Nanasato et al., 2013). Apart from increasing the number of hair roots produced, the use of younger cotyledons also helps to shorten the time needed for hairy root induction procedure (<1 month).

### *Rhizobium rhizogenes* K599 Strain Is Effective for Cucumber Hairy Root Induction

The hairy roots were effectively induced from the local cultivar Choka F1 using *R. rhizogenes* K599 strain. Bacterial strains have been known as an important factor for hairy root induction and transformation. In previous studies, different *R. rhizogenes* strains were used for *in vitro* hairy root induction in cucumber (Trulson et al., 1986; Shi et al., 2006; Shi and Lindemann, 2006; Anuar et al., 2011; JingLong et al., 2017) and the hairy root induction rates were variable depending on cucumber cultivars and transformation procedures. The hairy root induction rate of over 90% was reported by Shi et al. (2006) and Shi and Lindemann (2006) using the ATCC 15834 strain. Recently, *R. rhizogenes* K599 strain had been utilized for inducing hairy roots in cucumber with the frequency of 95% under *in vivo* conditions (Fan et al., 2020). In our study, the K599 strain could facilitate 100% infected cucumber explants to produce strong hairy roots, much higher than that of ATCC 15834 strain (83.3 ± 16.6%). The formation of callus instated of hairy roots by ATCC 15834 strain (Supplementary Figure S1) might be a reason to lower the number of hairy roots produced. The fewer callus formed from K599 inoculation were coupled with the better hairy root morphology. Therefore, the K599 strain is most suitable for hairy root induction and transformation of the local cultivar.

### A Convenient Tool to Assess the Activities of CRISPR/Cas9 Constructs in Cucumber

Hairy root systems have been used for genome editing of various plant species. For cucumber, the hairy root system has been applied for studying transgenic plant generation (Trulson et al., 1986), gene expression (Fan et al., 2020), promoter evaluation (Anuar et al., 2011), recombinant protein production (Shi and Lindemann, 2006), but not for genome editing yet. In this study, an efficient procedure for *in vitro* hairy root induction of a local cucumber cultivar (ChokaF1), which was successfully utilized for stable transformation (Hoang et al., 2021). This procedure was well performed for the expression of a reporter gene (*gus*) in a short time. The developed hairy root system was also effective for gene editing of cucumber using CRISPR/Cas9 construct. Targeted mutations of two endogenous genes (*CsbHLH66* and *CsbHLH82*) were induced accurately. Different indels of targeted genes were observed in tested hair roots including insertions, and larger and small deletions (Figure 7). All homozygous, heterozygous, and biallelic, as well as chimeric alleles could be obtained in the primary hairy roots (Figure 7). In addition, the targeting capability of different sgRNAs (single guide RNA) could be evaluated within 1 month, instated of several months or longer if using the transgenic plant approach. The targeted mutations induced in transgenic hairy roots were also observed in stable transgenic cucumbers (Chandrasekaran et al., 2016; Hu et al., 2017).

### *CsbHLH66* and *CsbHLH82* Genes Are Involved in Root Hair Performance of Cucumber

The root hair-less phenotype was observed in *Lotus japonicus* and characterized as a consequence of natural mutations in the *SjRHL1* gene (Karas et al., 2009). In *A. thaliana*, this phenotype was also found in the triple mutant of *AtLRL1*, *AtLRL2*, and *AtLRL3*, while sparse root hair phenotypes were caused by single or double mutations of these three genes (Karas et al., 2009). In our study, induced mutations in *CsbHLH82* resulted in the root hair spare phenotype (lines 15, 29), while simultaneous mutations of both *CsbHLH82* and *CsbHLH66* genes caused the root hair-less phenotype (lines 6, 10; Figures 7C,D). This result suggests that two bHLH transcription factors encoded by *CsbHLH82* and *CsbHLH66* are redundantly involved in regulating the root hair formation in cucumber. However, further research needs to be performed to characterize and confirm the contributions of *CsbHLH66*, *CsbHLH82*, and other *CsbHLH* genes in cucumber root hair formation at whole plant levels.

## Conclusion

In this study, an effective protocol for cucumber hairy root induction and transformation was developed for a local cucumber cultivar. This hairy root system has been successfully used to evaluate the expression of transgenes and the activities of CRISPR/Cas9 systems within 1 month. The obtained result obviously provides a potential tool for transgene expression and genome editing studies in cucumber as well as other *Cucumis* plants.

## Data Availability Statement

The original contributions presented in the study are included in the article/Supplementary Material, further inquiries can be directed to the corresponding authors.

## Author Contributions

PD and HC conceived and supervised the study. PD, DN, and TH designed the experiments, analyzed the data and prepared the manuscript. CN conducted the phylogenetic tree and designed the gRNAs. NL and HT performed CRISPR/Cas9 vector constructions. TH conducted cucumber hairy root transformation. DN performed genotyping and phenotyping of transgenic hairy roots. HC, Y-HM, and CN revised and proofread the manuscript. All authors contributed to the article and approved the submitted version.

## Funding

This research was supported by the National Foundation for Science and Technology of Vietnam (106.03-2019.11) and the Vietnam Academy of Science and Technology (ĐLTE00.10/20-21).

## Conflict of Interest

The authors declare that the research was conducted in the absence of any commercial or financial relationships that could be construed as a potential conflict of interest.

## Publisher’s Note

All claims expressed in this article are solely those of the authors and do not necessarily represent those of their affiliated organizations, or those of the publisher, the editors and the reviewers. Any product that may be evaluated in this article, or claim that may be made by its manufacturer, is not guaranteed or endorsed by the publisher.

## Supplementary Material

Supplementary Table S1 Primers used in this study.

Supplementary Figure S1 Cucumber hairy root induction by *Rhizobium rhizogenes* strain K599 and ATCC 15834. The photos were captured at 10 days after co-cultivation.

Supplementary Figure S2 GUS histochemical analysis of the herbicide resistant hairy roots. WT: non-transgenic hairy roots grown on the PPT free medium; 1–14: Hairy root lines survived and developed on the selection medium with PPT.

Supplementary Figure S3 Distribution of root hair phenotypes of tested cucumber hairy roots.

Supplementary Figure S4 The stability of induced mutations in propagated cucumber hairy roots. **(A)** Heteroduplex analysis of *CsbHLH66* gene. **(B)** Heteroduplex analysis of *CsbHLH82* gene. M: 100 bp DNA Ladder; WT: wild type; P6 and P10: The original hairy roots of lines 6 and 10; 6-1, 6-1-1, 10-1, 10-1-1: Propagated roots from lines 6 and 10 at the second and third generations. **(C,D)** Expanding targeted sequences of *CsbHLH82* and *CsbHLH66* genes from propagated roots of the hairy root lines 6 and 10. Amplicon sequencings were conducted by the Sanger method. The expected sequences of mutant alleles were denoted upper chromatograms. **(E)** Clone sequencing of *CsbHLH82* and *CsbHLH66* targeted regions of propagated cucumber hairy roots at the third generation. Red and blue letters indicate target and PAM sequences, respectively. Green letters indicate inserted nucleotides.
