# Supplementary material for: An Efficient Hairy Root System for Validation of Plant Transformation Vector and CRISPR/Cas Construct Activities in Cucumber (Cucumis sativus L.)
Source: Front Plant Sci. 2022 Feb 11;12:770062. doi: 10.3389/fpls.2021.770062 (PMC8874011; doi:10.3389/fpls.2021.770062)
Supplement: Supplementary Table S1 — Primers used in this study. [file Table_1.DOCX]

**Supplementary Table S1.** Primers used in this study

| **Primer’s name** | **Sequence of primers (5’ 🡪 3’)** | **Note** |
| --- | --- | --- |
| *Nco*I-*bar*-F | AAACCATGGATGAGCCCAGAACGACG | Amplifying of the *bar* gene |
| *Rsr*II-*bar*-R | AAACGGTCCGCTAGATTGTTGAGCAGATCTC |  |
| *bHLH66*-F | ACCTTCTCCTCCGACGATTTC | Genotyping of *CsBHLH66* (271 bp) |
| *bHLH66*-R | AGCCGTCTACTTCAACGACG |  |
| *bHLH82*-F | CAGATCCCTCCCTCTCACT | Genotyping of *CsBHLH82* (339 bp) |
| *bHLH82*-R | GTCGGCATTTCCAAGGGAG |  |
| *Bsa*I- *bHLH66*-gR | ATTATT**GGTCTC**GAAACCCGGAGTTGTATCGCCGACGCAA | Generation of *Bsa*I-*CsbHLH66* gRNA*-*gRNA scaffold*-U6* terminator*-Bsa*I |
| *bHLH66*-gR | AACCCGGAGTTGTATCGCCGACGCAATCACTACTTCGACTCTA |  |
| U6ter-*Bsa*I-R | ATATAT**GGTCTC**GATGGTATTGGTTTATCTCATC |  |
| *Bsa*I-*bHLH82*-gF | ATATAT**GGTCTC**GATTGGATGGGGTTAAGGTCCCAAGGTT | Generation of *Bsa*I*-U6* promoter-*CsbHLH82* gRNA-*Bsa*I |
| *bHLH82*-gF | TGGATGGGGTTAAGGTCCCAAGGTTTTAGAGCTAGAAATAGC |  |
| *Bsa*I-U6p-F | ATATAT**GGTCTC**GCCATTCGACTTGCCTTCCGC |  |

The underline sequences are target sequences.
