# Supplementary figures and images for: An Efficient Hairy Root System for Validation of Plant Transformation Vector and CRISPR/Cas Construct Activities in Cucumber (Cucumis sativus L.)
Source: Front Plant Sci. 2022 Feb 11;12:770062. doi: 10.3389/fpls.2021.770062 (PMC8874011; doi:10.3389/fpls.2021.770062)

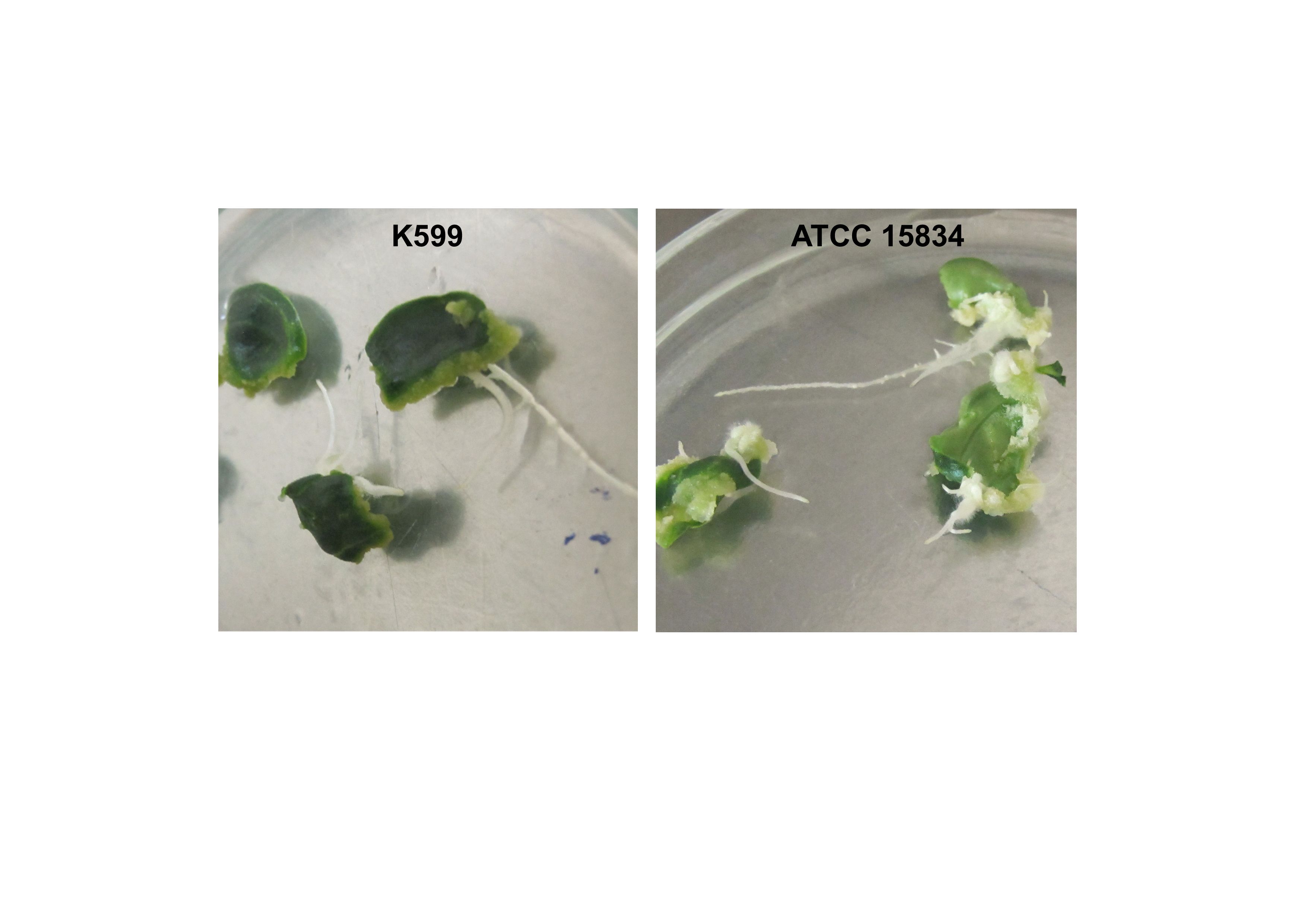

Supplement: Supplementary Figure S1 — Cucumber hairy root induction by Rhizobium rhizogenes strain K599 and ATCC 15834. The photos were captured at 10 days after co-cultivation. [file Image_1.JPEG]

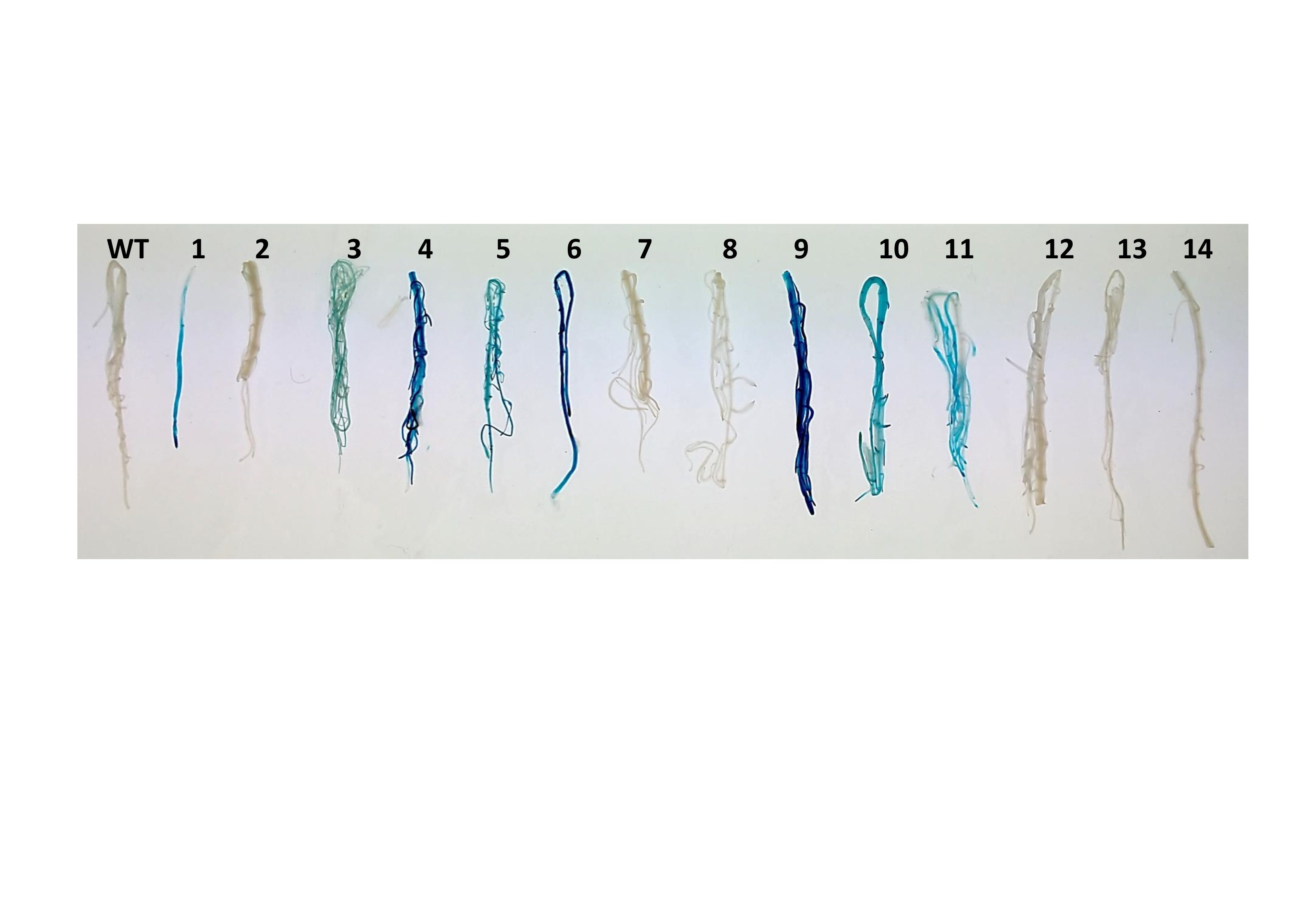

Supplement: Supplementary Figure S2 — GUS histochemical analysis of the herbicide resistant hairy roots. WT: non-transgenic hairy roots grown on the PPT free medium; 1–14: Hairy root lines survived and developed on the selection medium with PPT. [file Image_2.JPEG]

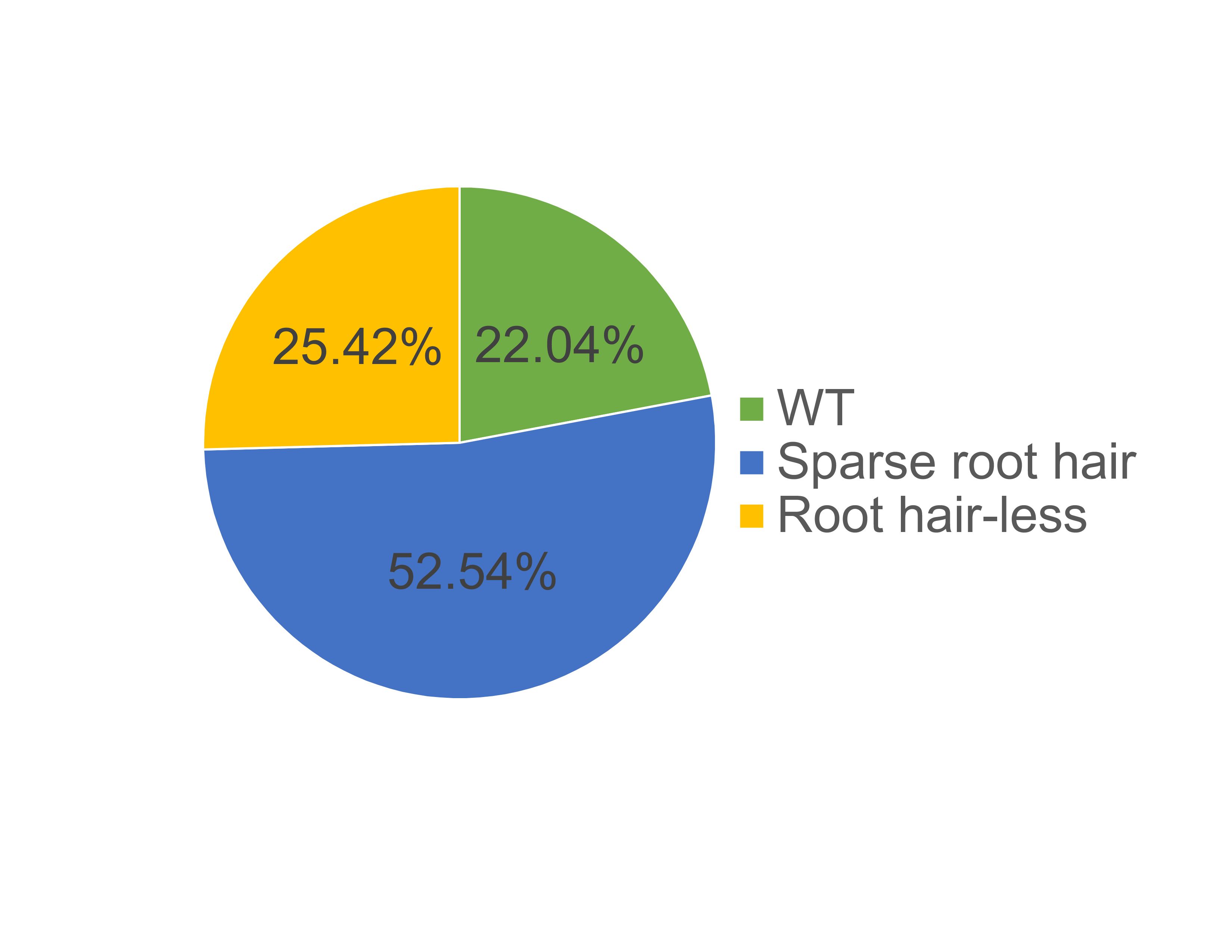

Supplement: Supplementary Figure S3 — Distribution of root hair phenotypes of tested cucumber hairy roots. [file Image_3.JPEG]

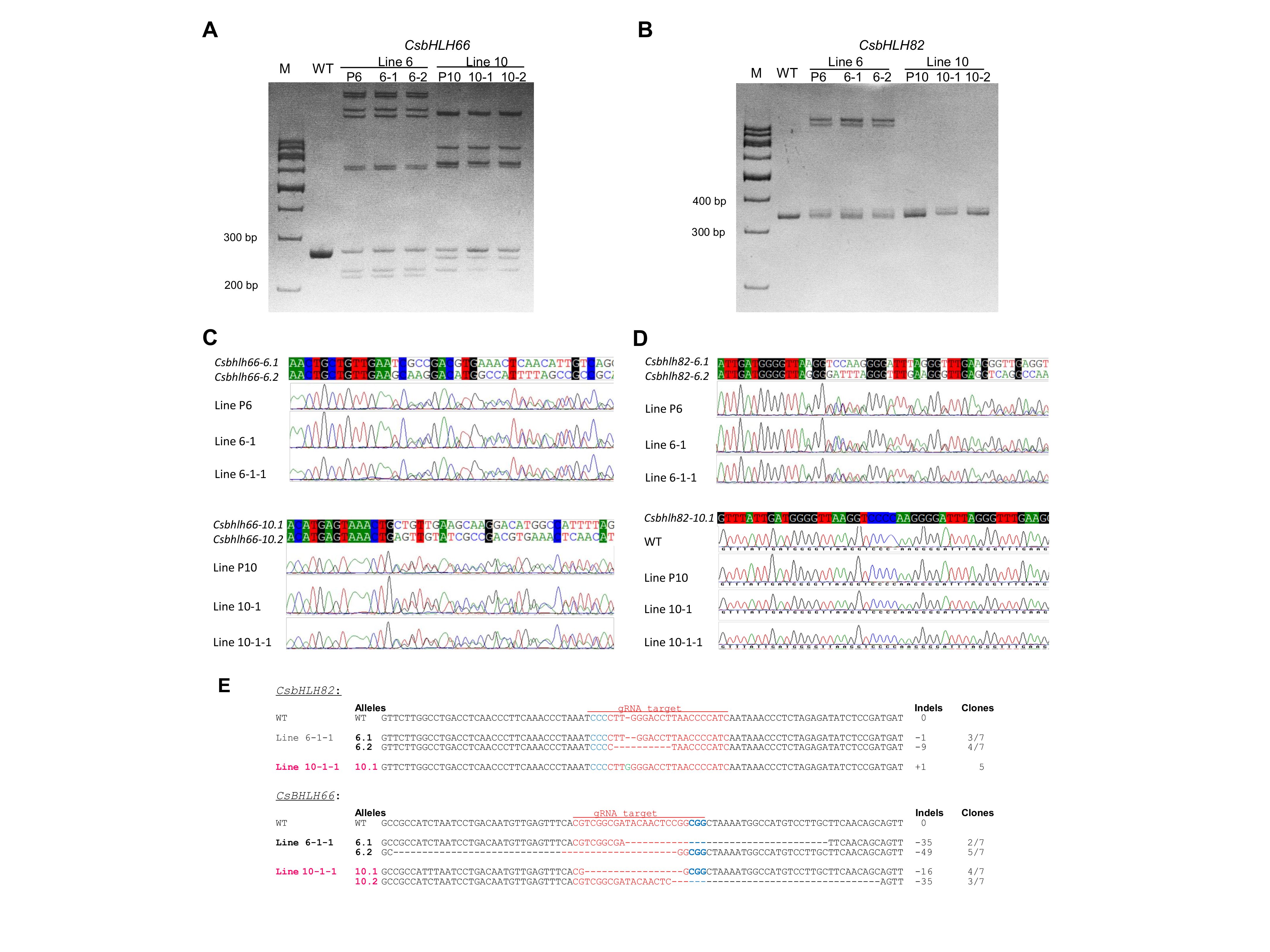

Supplement: Supplementary Figure S4 — The stability of induced mutations in propagated cucumber hairy roots. (A) Heteroduplex analysis of CsbHLH66 gene. (B) Heteroduplex analysis of CsbHLH82 gene. M: 100 bp DNA Ladder; WT: wild type; P6 and P10: The original hairy roots of lines 6 and 10; 6-1, 6-1-1, 10-1, 10-1-1: Propagated roots from lines 6 and 10 at the second and third generations. (C,D) Expanding targeted sequences of CsbHLH82 and CsbHLH66 genes from propagated roots of the hairy root lines 6 and 10. Amplicon sequencings were conducted by the Sanger method. The expected sequences of mutant alleles were denoted upper chromatograms. (E) Clone sequencing of CsbHLH82 and CsbHLH66 targeted regions of propagated cucumber hairy roots at the third generation. Red and blue letters indicate target and PAM sequences, respectively. Green letters indicate inserted nucleotides. [file Image_4.JPEG]
